# Functional autoantibodies and coronary microvascular obstruction in STEMI: a translational link between immune mechanisms and prognostic outcomes

**DOI:** 10.3389/fcvm.2026.1739236

**Published:** 2026-02-25

**Authors:** Laura Iop, Giovanni Civieri, Giacomo Bernava, Nicola Meynardi, Marta Vadori, Giulia Masiero, Marika Martini, Nicola Morat, Donatella Tansella, Sabino Iliceto, Emanuele Cozzi, Francesco Tona

**Affiliations:** 1 Cardiovascular Disease Modeling and Regenerative Medicine, Department of Cardiac, Thoracic, Vascular Sciences and Public Health, University of Padua, Padua, Italy; 2 Clinical Cardiology Unit, Department of Cardiac, Thoracic, Vascular Sciences and Public Health, University of Padua, Padua, Italy; 3 Transplantation Immunology Unit, Department of Cardiac, Thoracic, Vascular Sciences and Public Health, University of Padua, Padua, Italy; 4 Interventional Cardiology Unit, Department of Cardiac, Thoracic, Vascular Sciences and Public Health, University of Padua, Padua, Italy; 5 Heart Center, Mater Dei Hospital, Bari, Italy; 6 Department of Medicine and Surgery, LUM—Libera Università Mediterranea “Giuseppe Degennaro”, Bari, Italy

**Keywords:** AT1 receptor, autoantibodies, coronary microcirculation, endothelial dysfunction, ETA receptor, receptor antagonists, ST-elevation myocardial infarction

## Abstract

**Background:**

Despite timely primary percutaneous coronary intervention, coronary microvascular obstruction (CMVO) continues to limit myocardial reperfusion and worsen prognosis in patients with ST-elevation myocardial infarction (STEMI). Agonistic autoantibodies targeting the angiotensin II type 1 (AT1R) and endothelin-1 type A (ETAR) receptors have been associated with CMVO, but whether they directly contribute to microvascular injury remains unclear.

**Methods:**

We prospectively enrolled 287 STEMI patients and evaluated CMVO, left ventricular remodeling, and major adverse cardiovascular events during a median follow-up of 460 days. Immunoglobulins were isolated from a subset of patients with the highest AT1R-AA and ETAR-AA titers and from seronegative controls. Human cardiac microvascular endothelial cells were exposed to patient-derived or control immunoglobulins, with or without pharmacological receptor blockade.

**Results:**

Patients with higher autoantibody titers showed a greater prevalence of CMVO and worse clinical outcomes. *In vitro*, immunoglobulins from seropositive patients rapidly induced endothelial dysfunction, characterized by cytoskeletal disorganization, junctional disruption, endothelial activation, and increased mitochondrial oxidative stress. These alterations were most pronounced at 24 hours and progressed to reduced cell viability and increased cytotoxicity at 48 hours. Immunoglobulins from seronegative controls had no relevant effects. Blockade of AT1R and ETAR significantly mitigated endothelial injury and oxidative stress.

**Conclusions:**

Agonistic autoantibodies against AT1R and ETAR directly damage coronary microvascular endothelium and reproduce key features of CMVO observed in STEMI patients. These findings support a clinically relevant, immune-mediated mechanism of microvascular injury and suggest that receptor antagonism represents a biologically plausible, receptor-dependent mechanism warranting further investigation as a potential microvascular protective strategy in high-risk STEMI patients.

## Introduction

ST-elevation myocardial infarction (STEMI) remains a leading cause of morbidity and mortality worldwide. Although timely primary percutaneous coronary intervention (PPCI) restores epicardial coronary flow in most patients, effective myocardial reperfusion is frequently incomplete. Coronary microvascular obstruction (CMVO), often manifested as the no-reflow phenomenon, represents a major determinant of infarct size, adverse left ventricular remodeling, heart failure, and long-term mortality (1).

CMVO is a multifactorial process traditionally attributed to ischemia–reperfusion injury, distal embolization, endothelial dysfunction, inflammation, and intramyocardial hemorrhage. Despite advances in interventional techniques and adjunctive pharmacotherapy, CMVO remains difficult to predict and prevent, underscoring the need for a better understanding of its underlying mechanisms and for novel risk stratification tools (2).

In recent years, increasing evidence has highlighted a potential role of immune-mediated mechanisms in cardiovascular disease. In particular, circulating agonistic autoantibodies targeting G protein–coupled receptors, such as the angiotensin II type 1 receptor (AT1R) and the endothelin-1 type A receptor (ETAR), have been described in several vascular and inflammatory conditions. These autoantibodies act as functional receptor agonists, inducing sustained receptor activation and downstream pro-inflammatory and pro-fibrotic signaling (3–5).

Importantly, AT1R- and ETAR-directed autoantibodies have been detected in STEMI patients and have been associated with CMVO, adverse ventricular remodeling, and worse clinical outcomes (6). Beyond STEMI, these autoantibodies have been linked to chronic microvascular diseases, including resistant hypertension, systemic sclerosis, preeclampsia, and chronic allograft rejection (7, 8). However, these observations are largely associative. Whether these autoantibodies directly cause coronary microvascular endothelial injury, thereby contributing mechanistically to CMVO, remains unknown. In particular, direct experimental evidence linking autoantibody-mediated receptor activation to microvascular dysfunction in the human heart is lacking.

To address this critical gap, we developed a translational approach combining clinical data with a human cardiac microvascular endothelial cell model. We hypothesized that AT1R and ETAR agonistic autoantibodies directly impair coronary microvascular endothelial structure and function, and that pharmacological receptor blockade can mitigate this injury. By testing this hypothesis, we aimed to provide mechanistic insight into an immune-mediated pathway of CMVO and to explore its potential clinical and therapeutic implications in STEMI patients.

## Methods

### Study population

From January 2022, we prospectively enrolled 287 consecutive STEMI patients who presented at our Cardiology Intensive Care Unit, all treated with PPCI within 12 hours of symptom onset. Inclusion criteria were:

Typical ischemic chest pain
ST-segment elevation ≥0.1 mV in ≥2 contiguous ECG leads
Elevated high-sensitivity troponin (above the 99th percentile)

Patients with prior myocardial infarction, chronic atrial fibrillation, significant valvular disease, or poor echocardiographic imaging were excluded. A subsample of these patients underwent cardiac magnetic resonance imaging (CMR) within a median of 15 days post-STEMI to assess CMVO and was included in this study. Transthoracic echocardiography (TTE) was performed at 6 months to evaluate left ventricular remodeling (LVR). Median clinical follow-up for major adverse cardiovascular events (MACEs: cardiovascular death, nonfatal reinfarction, and heart failure hospitalization) was 460 days.

Blood samples for autoantibody testing were collected within 12 hours after PPCI revascularization and serum isolated. The study was approved by the institutional ethics committee (code number CESC 5478/AO/21), and informed consent was obtained from all patients. Detailed methods for the clinical study are described in the Supplementary Methods section.

### Isolation of IgGs and detection of AT1R-AAs and ETAR-AAs

AT1R-AAs and ETAR-AAs levels were determined in serum samples by ELISA. Seropositivity was defined as levels >10 U/mL (5), and patients were stratified accordingly. For the *in vitro* experiments, we intentionally focused on a small, extreme-phenotype subgroup as a proof-of-concept: 4 patients with double seropositivity for AT1R-AAs and ETAR-AAs and the highest titers within the cohort. This selection strategy was designed to maximize biological contrast while acknowledging the limited sample size. IgGs from these patients were purified (5) and normalized to 3.50 mg/mL. A 1:20 dilution was used in cell stimulation experiments (9–11). As a control group, 40 healthy subjects matched for age and sex were recruited from the general population (healthy blood donors). In particular, all healthy subjects were asymptomatic with no history of heart disease or endocrine disease. Exclusion criteria for all subjects included any of the following conditions: cerebral vascular disease, carotid artery bruit, peripheral bruit or abnormal pulse, history of angina or myocardial infarction, or hypertension requiring treatment. All participants had normal ECG at rest. Patients and healthy subjects came from the same geographic area (northeast Italy). In healthy subjects, the absence of coronary artery disease was evaluated by clinical history, physical examination, and ECG; 15% were seropositive for AT1R-AAs and/or ETAR-AAs. Four individuals with under-threshold autoantibody levels were used as negative controls for *in vitro* studies.

### *In vitro* cardiac microvascular endothelial cell model

After 24-hour serum starvation, primary healthy human cardiac microvascular endothelial cells (hcMVECs; 10,000 cells/cm²; commercial line) were incubated with seropositive STEMI patient IgGs or seronegative control IgGs (*n* = 4 each), with or without a combination of AT1R and ETAR inhibitors (Is):

Valsartan (10⁻⁵ M)
Bosentan (10⁻⁷ M) (12–14).

Acute exposure lasted 24 or 48 hours (respectively, T24 and T48), and possible variations were compared with pre-treatment (T0).

### *In vitro* readouts

Cell architecture and organization were investigated by phase contrast microscopy. MitoSOX™ Red (MR) was used to detect superoxide production as a marker of mitochondrial dysfunction. hcMVECs’ immunophenotype variations were investigated through VE-cadherin (CD144) endothelial marker expression, VCAM-1 (CD106) endothelial activation, and AT1R and ETAR distribution with targeting primary antibodies. Secondary antibodies (Alexa Fluor 555 or 488) and counterstaining (phalloidin-Atto 488 for F-actin and DAPI for nuclei) were used. Images were acquired using fluorescence microscopy. Morphometric analyses, including cell roundness, nuclear aspect ratio, and circularity, were performed with dedicated software. Cell viability and cytotoxicity were measured using commercial kits.

Detailed methods for the *in vitro* study are described in the Supplementary Methods section (Supplementary materials).

### Statistical analysis

For the *in vitro* study, continuous variables were expressed as median (IQR) or mean ± SD. Experimental data were normalized to T0 and reported as fold-changes. The Shapiro–Wilk test was used to assess normality. Normally distributed variables were analyzed by two-way ANOVA with Tukey's or Sidak's *post-hoc* tests.

For the clinical study, primary endpoints were CMVO, LVR, and MACEs. Mann–Whitney U, Wilcoxon, Kruskal–Wallis with Dunn's test were used to treat non-normally distributed data. Categorical data were analyzed with *χ*² or Fisher's exact tests as appropriate.

Multivariable stepwise logistic regression was used to assess differences between groups. A multivariable logistic regression model was constructed to identify independent predictors of CMVO. Based on clinical relevance, the core adjustment set included age, sex, ischemic time, and infarct location; diabetes mellitus was additionally included given its established association with microvascular dysfunction. Autoantibody positivity was forced into the model. To avoid overadjustment, infarct size—potentially downstream of microvascular injury—was not included in the primary model and was evaluated in sensitivity analyses together with other cardiovascular risk factors (hypertension, hypercholesterolemia, and smoking).

Variables with *p* < 0.05 were retained; those with *p* > 0.1 were excluded. All statistical tests were two-sided, with significance set at *p* < 0.05.

To complement *p*-values, effect sizes were calculated to quantify the magnitude of observed differences. For *in vitro* two-group comparisons, standardized mean differences were computed as Hedges' g with interpretation according to conventional thresholds (small ≈0.2, medium ≈0.5, large ≥0.8). For clinical outcomes, odds ratios (ORs) derived from multivariable logistic regression were reported as measures of effect size, together with 95% confidence intervals.

Analyses were performed with IBM SPSS v28 for clinical data and GraphPad Prism v8 for preclinical ones. All authors had full access to the data, contributed to, and approved the final manuscript.

## Results

### Cell morphometry and cytoskeletal organization

**Figure 1:**
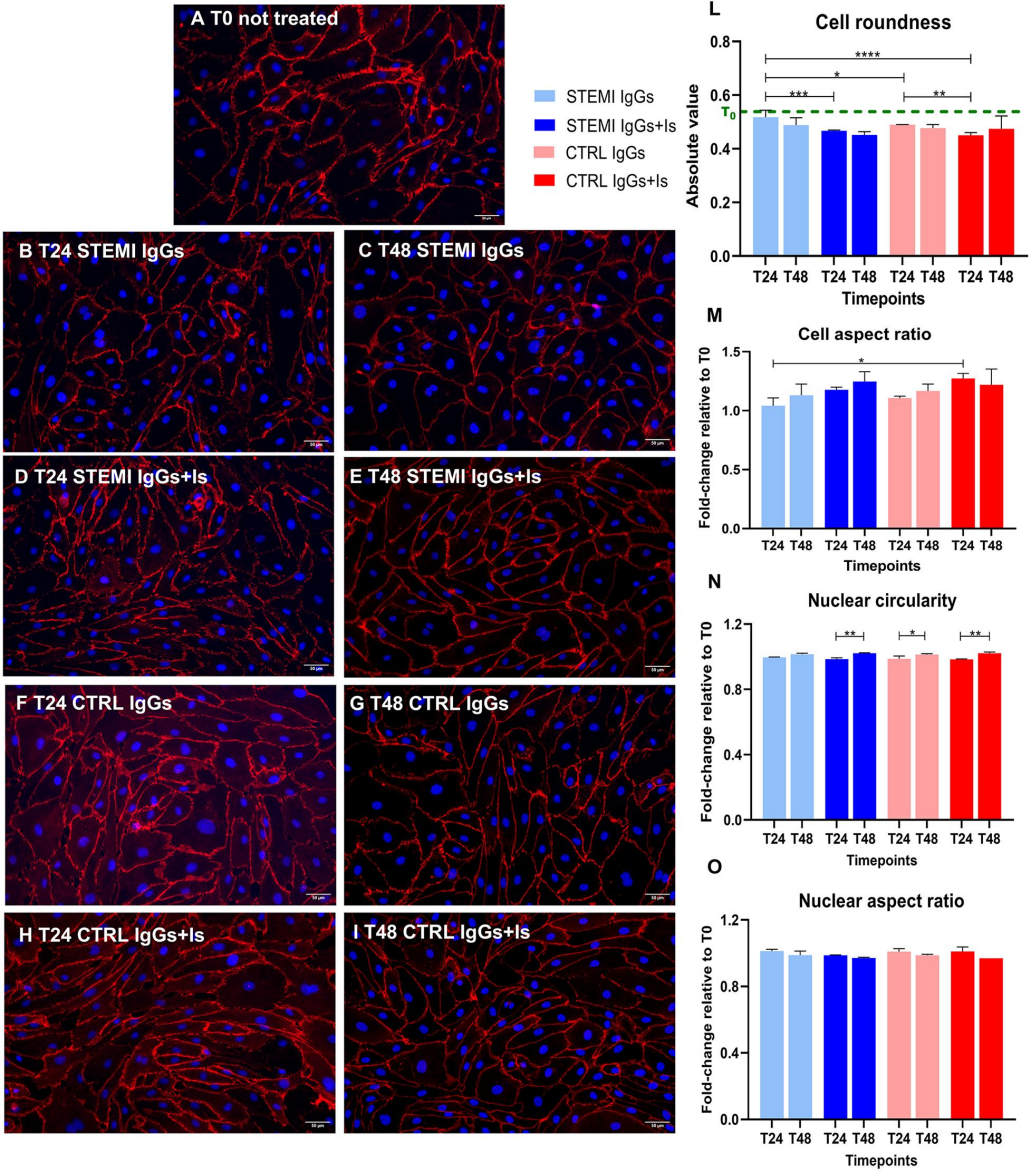
Effects of patient-derived IgGs on endothelial junctions and cell and nuclear morphology. Representative immunofluorescence images of hcMVECs stained for VE-cadherin (red, left panels) and nuclei (DAPI, blue). At baseline [**(A)**, T0], cells displayed intact junctions. STEMI IgGs induced cell size reduction and discontinued VE-cadherin expression at 24 hours **(B)** and 48 hours **(C)** receptor antagonists partially restored elongation **(D,E)**. Control IgGs likely preserved morphology **(F,G)**, with elongation under receptor blockade **(H,I)**. Cell morphometrics after STEMI IgG exposure revealed a decrease in cell aspect ratio but no change in roundness when compared to T0 **(L,M)**. Nuclear morphometrics **(N,O)** showed only minor time-dependent changes in circularity and no significant differences in aspect ratio, indicating preserved nuclear shape despite cytoplasmic injury. *p < 0.05, **p < 0.005. Scale bar = 50 μm.

**Figure 2:**
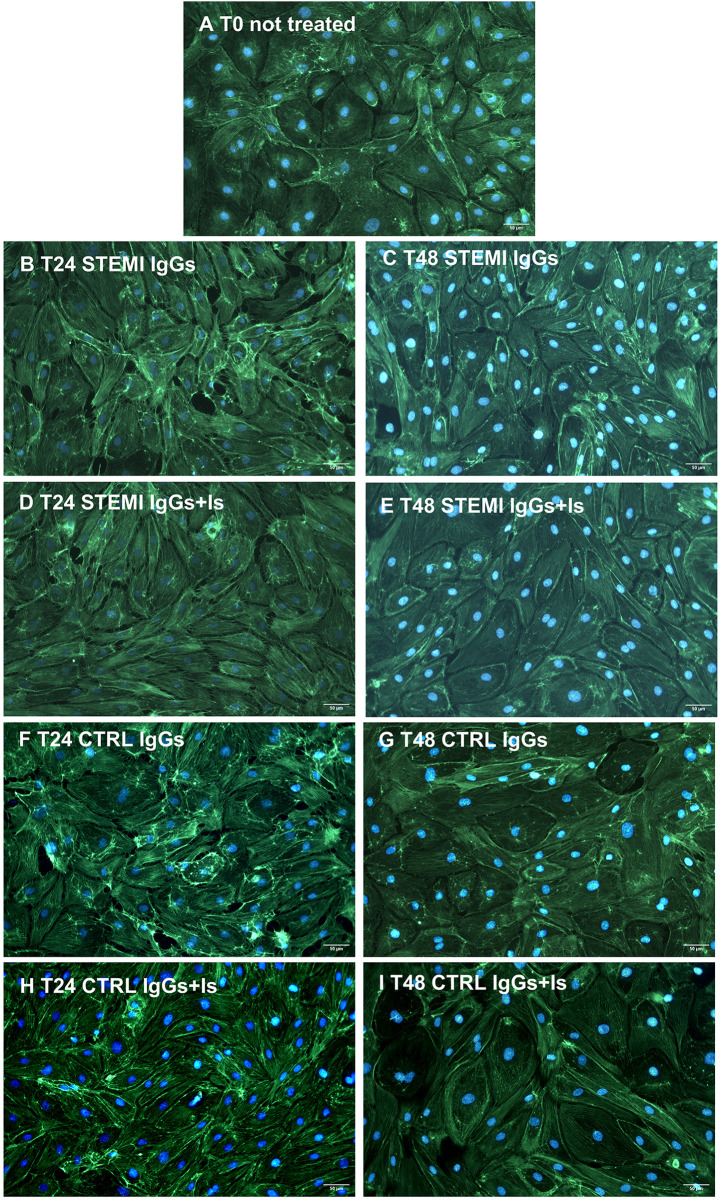
Effects of patient-derived IgGs on cytoskeletal organization. Representative immunofluorescence images of hcMVECs stained for F-actin (green) and nuclei (DAPI, blue). At baseline [**(A)**, T0], cells showed well-organized cytoskeleton. Exposure to STEMI IgGs caused F-actin filament disarray at 24 hours **(B)** and 48 hours **(C)**. The cytoskeleton appeared better organized in the presence of receptor antagonists **(D,E)**. No change in F-actin filament alignment was induced by control IgGs, with/without receptor blockade **(F–I)**. Scale bar = 50 μm.

At baseline, hcMVECs showed cobblestone morphology and aligned F-actin filaments (Figures 1A, 2A, and 1S). STEMI-derived IgGs induced size reduction, cytoskeletal disarray, and VE-cadherin discontinuity (Figures 1B,C, 2B,C), whereas control IgGs had minimal effects (Figures 1F,G, 2F,G). Receptor antagonists partially restored elongation and F-actin alignment (Figures 1D,E, 2D,E).

Morphometric measurements on VE-cadherin- and DAPI-stained cells evidenced a reduced cell aspect ratio between the minor and major axes after STEMI IgG conditioning, with no significant change in roundness compared to T0. Cell elongation was observed over time and significantly enhanced by receptor antagonism (Figures 1L,M and Supplementary Table S2). Nuclei of cells exposed to STEMI IgGs exhibited only minor time-dependent changes in circularity and no significant variations in aspect ratio (Figures 1N,O and Supplementary Table S2), with no consistent differences between treatments, indicating preserved nuclear integrity despite cytoplasmic alterations. Effect size analysis indicated large standardized effects for autoantibody-induced alterations in cell shape parameters, while nuclear morphometric changes were of smaller magnitude (Supplementary Table S4).

### Endothelial activation

**Figure 3:**
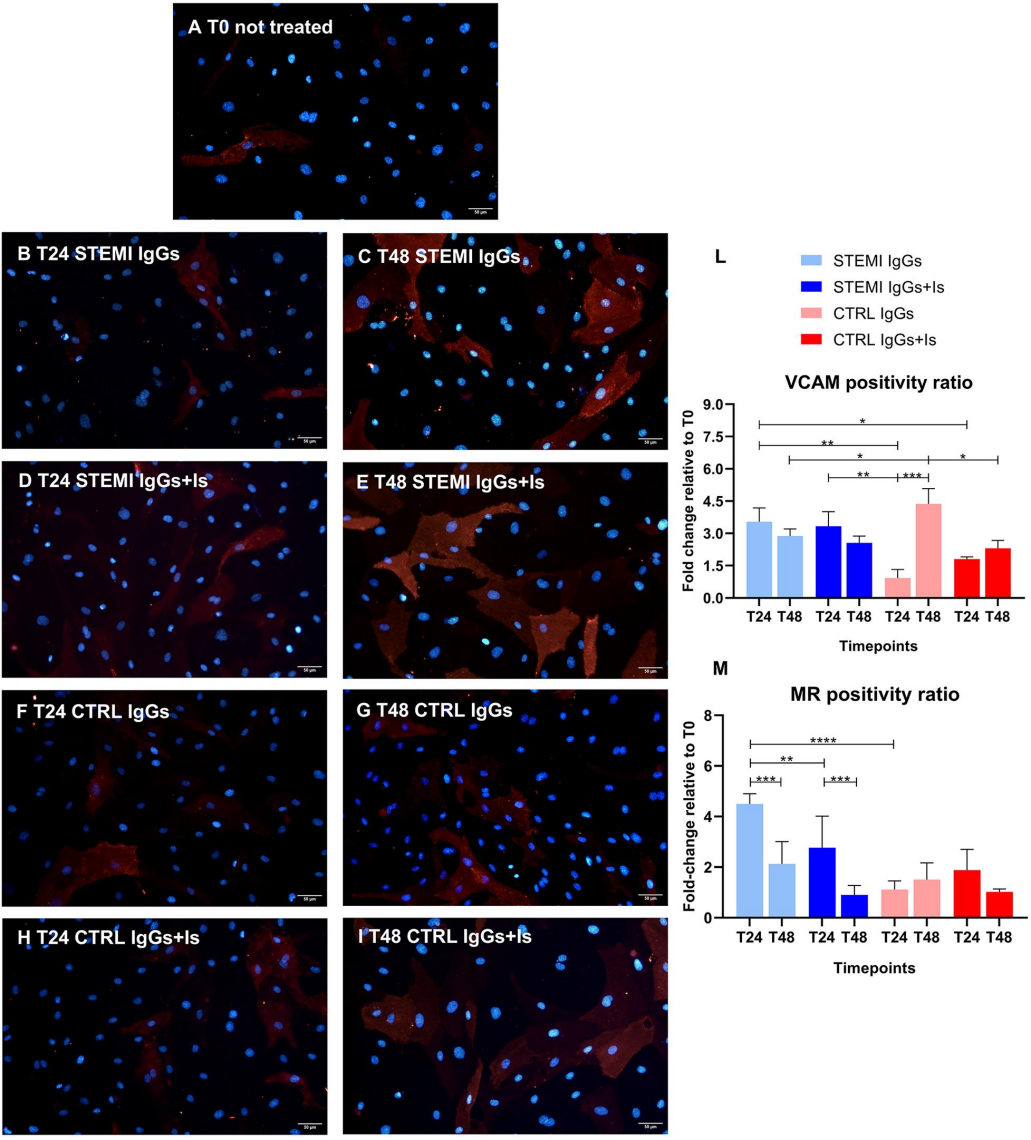
Autoantibody-induced endothelial activation (VCAM-1 expression) and mitochondrial dysfunction (superoxide production). Representative immunofluorescence images of hcMVECs stained for VCAM-1 (red, left panels) and nuclei (DAPI, blue). At baseline [**(A)**, T0], VCAM-1 expression was minimal. Exposure to STEMI IgGs increased VCAM-1 signal at 24 hours **(B)** and 48 hours **(C)**, particularly pronounced at T48. Receptor antagonists reduced this effect **(D,E)**. Control IgGs showed low expression **(F,G)**, further reduced under receptor blockade **(H,I)**. VCAM-positive cell quantification **(L)** confirmed a significant treatment–time interaction (*p* = 0.0009), with higher VCAM-1 positivity in STEMI IgG–treated cells at T24 (*p* = 0.0029) and attenuation by receptor inhibition. Mitochondrial superoxide production (MR positivity ratio) consistently increased after 24-h STEMI IgG exposure and was significantly abrogated by receptor antagonism. At 48 hours, the MR signal declined in the STEMI groups, consistent with oxidative injury and cell loss. Mitochondrial dysfunction signs were not observed in controls **(M)**. Two-way ANOVA confirmed significant effects of treatment, time, and their interaction. *p < 0.05; **p < 0.01; ***p < 0.001, ****p < 0.0001. Scale bar = 50 μm.

STEMI IgGs significantly increased VCAM-1 expression at 24 hours compared with controls (*p* = 0.0029), sustained through 48 hours (Figures 3B,C vs. 3F,G,L and Supplementary Table S2). This effect was quantitatively confirmed by VCAM-1–positive cell counts and supported by large standardized effect sizes. Antagonists attenuated this response (Figures 3D,E,H,I,L and Supplementary Table S2). Control IgGs induced only delayed VCAM-1 upregulation, counteracted by inhibitors (Figures 3F–L and Supplementary Table S2). Effect size analysis further supported the magnitude of the experimental findings, with large standardized effects for autoantibody-induced endothelial activation (Supplementary Table S4).

### Mitochondrial oxidative stress

Marked mitochondrial ROS generation was revealed at 24 hours in STEMI IgGs-treated cells vs. controls (*p* < 0.0001) and was alleviated by receptor blockade (Supplementary Table S2). ROS declined by 48 hours (Figure 3M and Supplementary Table S2), consistent with oxidative injury and cell loss. Consistently, effect size estimates demonstrated large standardized effects for autoantibody-induced mitochondrial oxidative stress (Supplementary Table S4).

### Receptor expression and localization

**Figure 4:**
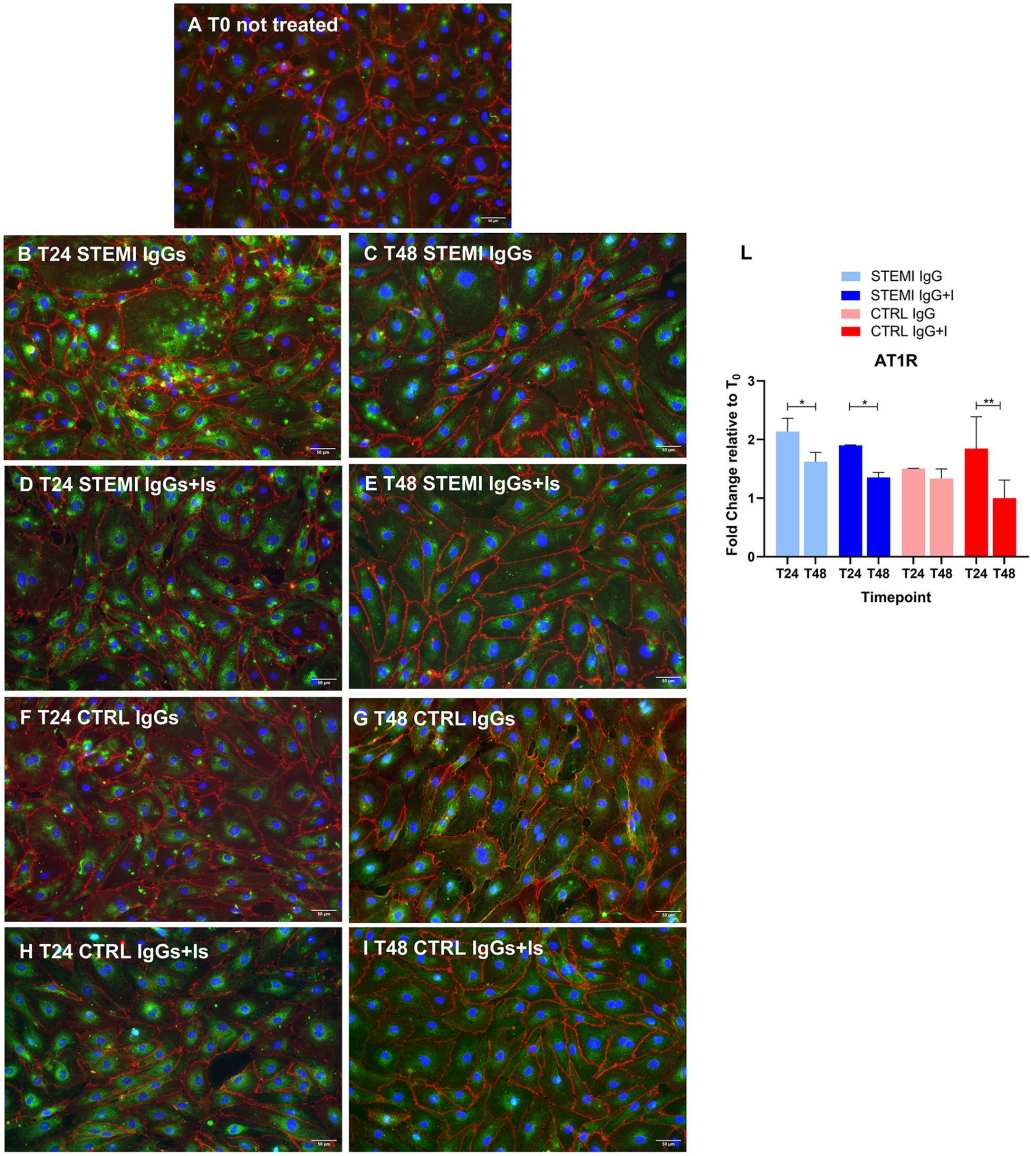
Autoantibody-induced redistribution of AT1R in hcMVECs. Representative immunofluorescence images showing AT1R (green) with VE-cadherin-positive cell endothelial membrane (red) and nuclear counterstaining (DAPI, blue). At baseline [**(A)**, T0], AT1R exhibited a diffuse membrane-associated distribution **(A)**. Exposure to STEMI IgGs induced marked perinuclear clustering of AT1R at 24 hours **(B)** and irregular punctate patterns consistent with cellular injury at 48 hours **(C)**. Co-treatment with valsartan and bosentan restored a more diffuse distribution **(D,E)**. Control IgGs induced only mild perinuclear changes **(F,G)**, with receptor antagonists promoting homogeneous peripheral expression **(H,I)**. Scale bar = 50 μm. A semi-quantitative assessment of AT1R immunofluorescence was expressed as a fold change relative to baseline (T0) **(L)**. At 24 hours, receptor fluorescence levels in cells treated with STEMI IgGs were higher than in controls and drugs-treated cells and remained elevated when compared to the same conditions at 48 hours. Two-way ANOVA revealed significant effects of time, treatment, and interaction, with post hoc analyses confirming a protective effect of valsartan and bosentan over time. All data are presented as mean ± SD. *p < 0.05.

**Figure 5:**
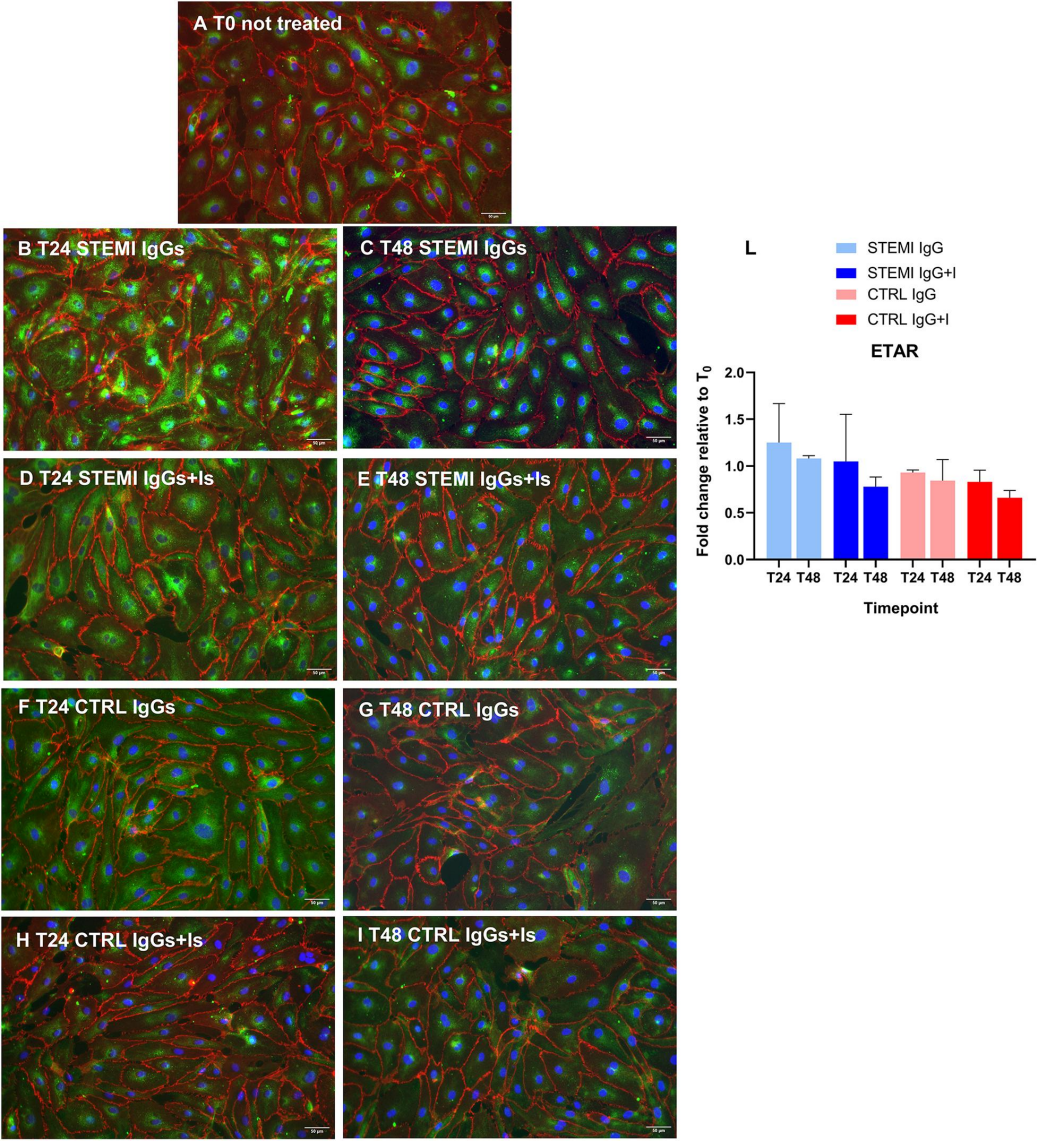
Autoantibody-induced redistribution of ETAR in hcMVECs. Representative immunofluorescence images showing ETAR (green) with VE-cadherin-positive cell endothelial membrane (red) and nuclear counterstaining (DAPI, blue). At baseline [**(A)**, T0], ETAR showed moderate perinuclear and cytoplasmic localization **(A)**. Exposure to STEMI IgGs induced marked perinuclear clustering of ETAR at 24 h **(B)** and irregular punctate patterns consistent with cellular injury at 48 h **(C)**. Co-treatment with valsartan and bosentan restored a more diffuse distribution **(D,E)**. Control IgGs induced only mild perinuclear changes **(F,G)**, with receptor antagonists promoting homogeneous peripheral expression **(H,I)**. Scale bar = 50 μm. A semi-quantitative assessment of ETAR immunofluorescence was expressed as a fold change relative to baseline (T0) **(L)**. At 24 h, receptor fluorescence levels in cells treated with STEMI IgGs were higher than in controls and drugs-treated cells and remained elevated when compared to the same conditions at 48 h. Two-way ANOVA revealed significant effects of time, treatment, and interaction, with post hoc analyses confirming a protective effect of valsartan and bosentan over time. All data are presented as mean ± SD. *p < 0.05.

Both AT1R and ETAR redistributed from more diffuse (Figure 4A for AT1R and 5A for ETAR) to perinuclear clusters under STEMI IgG exposure, an effect partially normalized by antagonists (Figures 4B,C vs. 4D,E for AT1R and Figures 5B,C vs. 5D,E for ETAR). This effect was quantitatively confirmed (Figures 4L, 5L) and supported by large standardized effect sizes. Control IgGs induced only mild changes (Figures 4F,G for AT1R and Figures 5F,G for ETAR), abrogated by receptor antagonism (Figures 4H,I for AT1R and 5H,I for ETAR). Consistent with the quantitative findings, autoantibody exposure induced a marked redistribution of AT1R and ETAR from a diffuse to a perinuclear pattern, which was largely prevented by receptor blockade. While this endpoint was assessed semi-quantitatively (Figures 4L, 5L), its magnitude was concordant with the large effect sizes observed for endothelial activation, oxidative stress, and cytotoxicity (Supplementary Table S4).

### Cell viability and cytotoxicity

**Figure 6:**
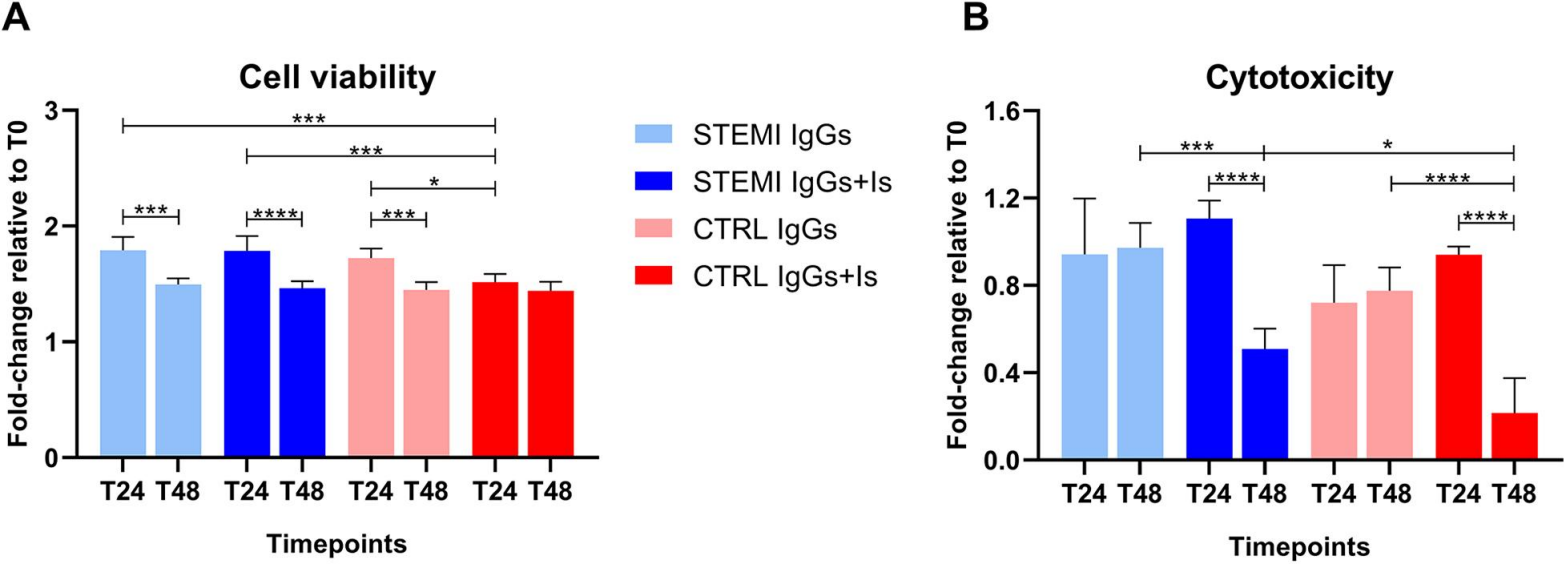
Effects of patient-derived IgGs on hcMVEC viability and cytotoxicity. Cell viability was assessed using the MTS assay and expressed as a fold change relative to baseline (T0). At 24 hours, hcMVECs exposed to STEMI IgGs showed reduced viability compared with controls, whereas receptor antagonists partially improved survival. By 48 hours, viability declined significantly in both STEMI IgGs and STEMI IgGs + Is groups, consistent with progressive cell injury. Control IgGs supported greater proliferation, while receptor blockade reduced this effect. Two-way ANOVA revealed significant effects of time, treatment, and their interaction, with *post-hoc* analysis confirming a time-dependent decline in viability in the STEMI groups and controls, but not in Control IgGs + Is **(A)** cytotoxicity was assessed by LDH release and expressed as a fold change relative to baseline (T0). At 24 hours, LDH levels remained close to baseline across groups. At 48 hours, STEMI IgGs induced a significant increase in LDH, consistent with progressive cell membrane damage, whereas receptor antagonists significantly reduced this effect. Control IgGs induced only modest LDH release, further attenuated by antagonists. Two-way ANOVA revealed significant effects of time, treatment, and their interaction, with *post-hoc* analyses confirming a protective effect of valsartan and bosentan over time **(B)** All data are presented as mean ± SD. *p < 0.05; ***p < 0.001; ****p < 0.0001.

Viability decreased significantly from 24 to 48 hours in STEMI IgGs-treated cells (*p* = 0.0003), with partial protection by antagonists (Figure 6A and Supplementary Table S2). LDH release confirmed increased cytotoxicity at 48 hours with STEMI IgGs, significantly reduced by valsartan/bosentan (Figure 6B and Supplementary Table S2). Effect size analysis showed large effects for reductions in cell viability and increases in cytotoxicity, as well as for the protective impact of receptor blockade (Supplementary Table S4).

### Clinical study

As detailed in the Supplementary Results, findings from the patient cohort corroborated the mechanistic observations, showing that higher autoantibody titers were associated with a greater prevalence of CMVO and poorer outcomes. (Supplementary Table S3). Moreover, the association between autoantibody positivity and major clinical characteristics was specifically analysed to assess potential confounding and is also reported in Supplementary Table S3. The data that support the findings of this study are available from the corresponding author upon request. The strength of the associations observed in the clinical cohort is summarized in Supplementary Table S5. In multivariable logistic regression analysis, autoantibody positivity remained independently associated with CMVO after adjustment for age, sex, ischemic time, infarct location, and diabetes mellitus. Sensitivity analyses including additional cardiovascular risk factors and infarct size yielded consistent results (Supplementary Table S6).

## Discussion

Our findings provide proof-of-concept evidence that autoantibodies targeting AT1R and ETAR can directly induce coronary microvascular endothelial injury in STEMI. Exposure of healthy human cardiac microvascular endothelial cells to IgGs from double-seropositive patients resulted in morphological, inflammatory, and oxidative alterations that mirror key features of coronary microvascular obstruction *in vivo* (15–17). Importantly, these effects were attenuated by receptor-specific antagonists, identifying a mechanistically defined and potentially actionable therapeutic target.

Despite advances in procedural techniques and adjunctive pharmacotherapy, coronary microvascular obstruction remains difficult to predict and treat after STEMI, underscoring persistent gaps in the understanding of its pathophysiology (18). In recent years, functional autoantibodies have emerged as potential contributors to vascular injury. Autoantibodies targeting AT1R and ETAR have been reported in systemic sclerosis, preeclampsia, transplantation, and viral infections (19). Unlike neutral binders, these antibodies act as receptor agonists, inducing sustained activation of G protein–coupled signaling pathways (20). This persistent stimulation disrupts physiological regulation and promotes endothelial dysfunction, inflammation, and fibrosis (21). Within this context, our findings show that autoantibodies from STEMI patients with CMVO exert similar pathogenic effects in the coronary microcirculation.

Marked disruption of endothelial architecture was observed within 24 hours, with reduced cell size and disorganization of cytoskeletal F-actin. Loss of VE-cadherin continuity further indicated junctional disassembly and endothelial detachment. These changes are consistent with impaired barrier integrity and interstitial oedema described in regions of coronary microvascular obstruction. Importantly, receptor antagonism largely reversed these alterations, restoring cell elongation and cytoskeletal alignment. This finding supports a receptor-mediated mechanism underlying endothelial injury.

In addition to structural alterations, endothelial functional activation was evident. Exposure to STEMI IgGs induced robust VCAM-1 expression, a key adhesion molecule involved in leukocyte recruitment and microvascular plugging (22, 23). VCAM-1 expression peaked at 24 hours and subsequently declined, indicating a transient and time-dependent inflammatory response. Notably, the early peak observed with STEMI IgGs, compared with the delayed response in controls, mirrors the inflammatory burst reported during reperfusion in STEMI patients (24).

At 24 hours, mitochondrial ROS generation was markedly increased, consistent with sustained receptor stimulation by autoantibodies. This pro-oxidant state aligns with mechanisms implicated in ischemia–reperfusion injury and chronic vascular remodeling (25, 26). By 48 hours, ROS levels declined, in parallel with reduced cell viability and increased cytotoxicity. Together, these findings suggest a transition from acute oxidative stress to overt cell injury. Receptor antagonists attenuated both ROS production and LDH release, supporting a receptor-dependent mechanism of damage.

The abnormal perinuclear clustering of AT1R and ETAR observed in our model is consistent with altered receptor trafficking and sustained signaling. Similar receptor redistribution has been described in conditions of chronic GPCR overstimulation (27) and is thought to contribute to endothelial dysfunction by limiting physiological receptor desensitization. Importantly, this pattern was mitigated by receptor antagonists, supporting a receptor-dependent mechanism.

Collectively, the *in vitro* cellular alterations observed in this study recapitulate key pathological features of coronary microvascular obstruction *in vivo*. Disruption of endothelial architecture, inflammatory activation, mitochondrial oxidative stress, and loss of cell viability reflect hallmark changes of the post-infarction microvasculature during no-reflow (28).

Consistent with these experimental findings, clinical data from the patient cohort used for IgG extraction supported the proposed mechanism. Autoantibody positivity was associated with the presence of CMVO on cardiac imaging and with a higher incidence of major adverse cardiovascular events, including reinfarction, heart failure, and adverse ventricular remodeling during follow-up.

The descriptive comparisons reported in Supplementary Table S3 and the multivariable logistic regression analysis shown in Supplementary Table S6 are fully concordant. Baseline demographic and clinical characteristics were well balanced across autoantibody serostatus groups, whereas CMVO showed a strong and graded increase with autoantibody positivity. Importantly, this association remained independent in multivariable analysis after adjustment for established clinical and procedural determinants of CMVO, including infarct location and ischemic time. Variables that did not differ across groups in the descriptive analysis did not emerge as independent predictors in the adjusted model, supporting internal consistency and minimizing the likelihood of residual confounding.

Importantly, endothelial dysfunction was not observed when cells were exposed to IgGs from seronegative patients, paralleling the lower risk of CMVO and adverse outcomes documented in this clinical subgroup.

These findings have relevant translational implications. First, they support the potential inclusion of AT1R-AA and ETAR-AA screening in risk stratification strategies for STEMI patients, as elevated autoantibody titers may help identify individuals at higher risk of CMVO and inform early management decisions (29). Second, the receptor-dependent nature of the observed injury highlights targeted therapeutic opportunities. Angiotensin receptor blockers and endothelin receptor antagonists, already approved for cardiovascular indications, could be explored as peri-procedural strategies to mitigate microvascular injury during reperfusion (30). Third, our results reinforce the broader contribution of autoantibody-mediated mechanisms to cardiovascular disease, positioning functional autoantibodies at the interface between immunology and cardiology (31).

## Study limitations

Despite its strengths, this study has several limitations. First, it represents a proof-of-concept validation of a human cardiac microvascular *in vitro* model based on a limited number of extreme-phenotype STEMI patients, which strengthens mechanistic inference but limits generalizability. Future studies will extend this model to milder autoantibody phenotypes and larger sample sizes to assess dose-dependent effects and the impact of single seropositivity.

Second, demographic and clinical variables such as age, sex, comorbidities, infarct location, and concomitant therapies were not specifically controlled, although the use of human cardiac microvascular cells provides a suitable platform to explore age- and sex-related immune mechanisms in future investigations.

Third, receptor specificity was confirmed pharmacologically, but complementary genetic approaches were not employed, and downstream signaling pathways were not fully characterized. Fourth, while the *in vitro* model offers high mechanistic resolution and translational relevance, it cannot fully recapitulate the *in vivo* environment. *In vivo* validation, including passive autoantibody transfer in animal models, will therefore be necessary to confirm causality and advance therapeutic hypotheses (32).

Finally, the relative contribution of AT1R vs. ETAR signaling could not be dissected in the present study, as single receptor antagonism was not systematically evaluated.

## Conclusions

By integrating clinical associations with a human cardiac microvascular–specific experimental model, this study establishes a translational framework linking functional autoantibodies to microvascular injury and prognosis in STEMI.

This study provides mechanistic evidence that elevated AT1R-AA and ETAR-AA titers in STEMI patients contribute to coronary microvascular dysfunction through sustained receptor activation. Autoantibody exposure induced cytoskeletal disruption, inflammatory activation, mitochondrial oxidative stress, and reduced endothelial viability, reproducing key features of coronary microvascular obstruction. Importantly, these effects were attenuated by pharmacological receptor blockade, indicating a potentially actionable therapeutic target.

From a clinical perspective, autoantibody screening may improve risk stratification and support more personalized strategies to preserve microvascular integrity during and after myocardial infarction. More broadly, our findings highlight the pathogenic role of functional autoantibodies in cardiovascular disease and support their integration into future translational and clinical research frameworks.

## Data Availability

The raw data supporting the conclusions of this article will be made available by the authors, without undue reservation.
