# Supplementary material for: Functional autoantibodies and coronary microvascular obstruction in STEMI: a translational link between immune mechanisms and prognostic outcomes
Source: Front Cardiovasc Med. 2026 Feb 25;13:1739236. doi: 10.3389/fcvm.2026.1739236 (PMC12977973; doi:10.3389/fcvm.2026.1739236)
Supplement: Supplementary file 1 [file Datasheet1.pdf]

Supplementary materials

Functional Autoantibodies and Coronary Microvascular Obstruction in STEMI: A Translational Link Between Immune Mechanisms and Prognostic Outcomes

Laura Iop, PhD[a]; Giovanni Civieri, MD[b]; Giacomo Bernava, BSc[a]; Nicola Meynardi, MD[b]; Marta Vadori, PhD[c]; Giulia Masiero, MD[d]; Marika Martini, MD[b]; Nicola Morat, MD[b]; Sabino Iliceto, MD[e,f]; Emanuele Cozzi, MD, PhD[c]; Francesco Tona, MD, PhD[b]

[a]Cardiovascular Disease Modeling and Regenerative Medicine, Department of Cardiac, Thoracic, Vascular Sciences and Public Health, University of Padua, Padua, Italy.

[b]Clinical Cardiology Unit, Department of Cardiac, Thoracic, Vascular Sciences and Public Health, University of Padua, Padua, Italy.

[c]Transplantation Immunology Unit, Department of Cardiac, Thoracic, Vascular Sciences and Public Health, University of Padua, Padua, Italy.

[d]Interventional Cardiology Unit, Department of Cardiac, Thoracic, Vascular Sciences and Public Health, University of Padua, Padua, Italy.

[e]LUM - Libera Università Mediterranea "Giuseppe Degennaro"; Bari, Italy.

[f]Heart Center, Mater Dei Hospital; Bari, Italy.

Supplementary Methods

Preclinical modeling

Isolation of IgGs and Detection of AT1R-AAs and ETAR-AAs

Serum samples were collected within 12 hours of PPCI and stored at –80 °C. AT1R-AAs and ETAR-AAs levels were determined by ELISA (positivity >10 U/mL; CellTrend, Luckenwalde, Germany). (1)

Total IgGs were purified using the MelonTM gel IgG spin purification kit according to the manufacturer's instructions (Thermo Scientific, Waltham, MA, USA). Briefly, sera collected from selected patients (n=4) were diluted 1:10 v/v in Melon Gel Purification Buffer. Melon gel slurry (500 µL) was dispensed into spin columns placed in a microcentrifuge tube, subsequently centrifuged at 4,000 x g for 1 min. The columns were equilibrated with two washes of purification buffer, followed by a 10-second pulse centrifugation. Diluted serum (500 µL) was applied to the columns and incubated at room temperature for 5 min with end-over-end mixing before centrifugation at 4,000 x g for 1 min. To assess the purity of the extracted samples, SDS-PAGE electrophoresis was performed, followed by Coomassie staining of the gels. IgG concentration was quantified using Bradford Protein Assay, according to the manufacturer's instructions (AppliChem GmbH, Darmstadt, Germany).

To expose cells to the same total IgG concentration, independent of the extraction yield from the patient's serum, normalization to 3.50 mg/ml was carried out, and a further 1:20 dilution was used in stimulation experiments (final IgG concentration: 0.175 mg/ml). Such a total IgG concentration was chosen with reference to previously established in vitro endothelial models of autoimmunity in the context of other pathological conditions, such as pre-eclampsia and systemic sclerosis. (2-4)

Serum from matched healthy donors (n=40) was isolated as aforementioned, and samples with under-threshold AT1R-AAs and ETAR-AAs titers (n=4) were used as controls. Before modeling experiments, total IgGs extracted from healthy donors were also normalized to 3.5 mg/ml and used at a final concentration of 0.175 mg/ml.

In Vitro Cardiac Microvascular Endothelial Cell Model

Primary human cardiac microvascular endothelial cells (hcMVECs) were obtained commercially (Promocell, Heidelberg, Germany; cat. n° C-12285, batch n° 447Z026.7). Following the manufacturer's datasheet, cells were harvested from the heart ventricles of a single donor, and their endothelial phenotype was characterized by immunofluorescence, with positive expression of typical endothelial markers, such as CD31, von Willebrand factor, and Dil-Ac-LDL uptake, and negative expression of alpha smooth muscle actin. hcMVECs were cultured in complete EGM2-MV medium (Lonza, Milan, Italy), passaged at 80–85% confluence, and seeded at p8 at a density of 10,000 cells/cm² in 8-well chamber slides (Millipore, Merck, Milan, Italy). During expansion before starting the modeling experiments, cells were progressively characterized by immunofluorescence for the expression of PECAM (CD31), VE-cadherin, AT1R, and ETAR (methodology described in the following sections).

After 24-hour serum starvation (1% fetal bovine serum, Lonza), cells were treated with patient or control IgGs (n=4 each), with or without a combination of AT1 and ETA inhibitors (I):

- Valsartan (10^{-5} M; PHR1315, Merck)
- Bosentan (10^{-7} M; Roche, Basel, Switzerland).

Drug concentrations were selected based on previous literature on in vitro models of other endothelial cell types under different pathological conditions, such as pre-eclampsia and systemic sclerosis. (5-7)
Cells were exposed acutely for 24 or 48 hours to counteract potential bias introduced by medium changes (necessary after 48 hours for these cells).
No treatment (T0) was used for comparison.

Cellular Readouts

- **Cell architecture and organization**

Cell architecture and organization were investigated using phase contrast microscopy. Observation and image collection were performed with an inverted microscope (DMIL LED, Leica, Wetzlar, Germany).

- **Morphometrics**

Image processing, automatic cell counting, and morphometric analysis were performed using Leica LAS X Office 1.4.7 (Leica Mycrosystems, Wetzlar, Germany) and Fiji 1.54p. (8)
Regarding the morphometric evaluation, the metrics employed are defined as follows:

i. Cell roundness is a shape descriptor of the total cell, accounting for the area and the major axis as evidenced by VE-cadherin-marked endothelial cell membrane, as follows:

$$Cell\ roundness = \frac{4 \times Cell\ area}{\pi \times Cell\ major\ axis^2}$$

A value of 1 indicates a perfect cell circle. As the value approaches 0, it means an increasingly elongated cell shape. This descriptor was chosen for its insensitivity to irregular boundaries, such as the endothelial cell membranes marked by VE-cadherin.

ii. Cell aspect ratio is a shape descriptor of the whole cell, defined as the ratio of the major axis to the minor axis of each VE-cadherin-positive cell:

$$Cell\ aspect\ ratio = \frac{Cell\ major\ axis}{Cell\ minor\ axis}$$

A value of 1 means that the analyzed object is symmetric. Values >1 indicate cell elongation.

iii. Nuclear circularity is a shape descriptor of the nucleus, accounting for its area and perimeter evidenced by DAPI counterstaining, and it is defined as:

$$Nuclear\ circularity = \frac{4\pi \times Nuclear\ area}{Nuclear\ perimeter^2}$$

A value of 1 indicates a perfect circle. As the value approaches 0, it means an increasingly elongated nuclear shape.

iv. Nuclear aspect ratio is a shape descriptor of the nucleus, defined as the ratio of the major axis to the minor axis of each DAPI-positive cell organelle:

$$Nuclear\ aspect\ ratio = \frac{Nuclear\ major\ axis}{Nuclear\ minor\ axis}$$

A value of 1 means that the analyzed object is symmetric. Values >1 indicate nuclear elongation. (9,10)

- **Indirect immunofluorescence**

The hcMVEC immunophenotype was studied before and after treatment on 4% paraformaldehyde-fixed cells (Merck) by investigating the expression of typical endothelial markers involved in the cell membrane (PECAM (CD31) and VE-cadherin (CD144)), activation (VCAM-1 (CD106)), as well as endothelin 1 and angiotensin II binding (AT1R and ETAR) with targeting primary antibodies, as follows:

- Anti-PECAM (CD31), rabbit polyclonal antibody (Abbiotec, California, USA), diluted 1:25 (final concentration: 40 μg/mL);
- Anti-VE-cadherin (CD144), mouse monoclonal antibody (Invitrogen, Massachusetts, USA), diluted 1:50 (final concentration: 10 μg/mL);
- Anti-VCAM-1 (CD166), mouse monoclonal antibody (Invitrogen, Massachusetts, USA), diluted 1:5 (final concentration: 40 μg/mL);
- Anti-AT1R, rabbit polyclonal antibody (Invitrogen, Massachusetts, USA), diluted 1:500 (final concentration: 2 μg/mL);
- Anti-ETAR, rabbit polyclonal antibody (Invitrogen, Massachusetts, USA), diluted 1:500 as recommended by the vendor.

Secondary antibodies (Goat anti-mouse IgG (H+L) Alexa Fluor™ 555 (Invitrogen), 4 μg/mL, and Goat anti-rabbit IgG (H+L) Alexa Fluor™ 488 (Invitrogen), 4 μg/mL) were used. Antibody dilution was operated in 3% bovine serum albumin.

Cytoskeletal architecture was examined by co-staining with phalloidin conjugated to Atto 488 (0,15 μg/mL, Merck). DAPI-containing medium (Fluoroshield, Merck) was utilized to mount the cell slides.

Observation and image acquisition (region of interest area: 737,04 μm x 491,28 μm) were performed using inverted fluorescence microscopy (Leica).

For VCAM, AT1R, and ETAR, a semiquantitative assessment was performed using ImageJ software. In collected images, VCAM-positive cells (green fluorescence) were counted for each condition over the total number of cells (contrast phase) in 3 regions of interest (area of 737,04 μm x 491,28 μm). The mean ratio was

normalized to baseline (T0) to compare fold changes. Regarding AT1R and ETAR, the integrated fluorescence density per cell for each experimental condition was assessed, and the values were normalized as fold changes relative to the T0 condition.

- **Mitochondrial Dysfunction**

MitoSOX™ Red (MR; ThermoFisher, Waltham, MA, US) was used at a working concentration of 250 nM to detect mitochondrial superoxide production in living cells. Upon incubation as specified by the manufacturer, superoxide production was investigated within 2 hours by inverted fluorescence microscopy (Leica). In collected images, MR-positive cells (red fluorescence) were counted for each condition over the total number of cells (contrast phase) in 3 regions of interest (area of 737,04 μm x 491,28 μm). The mean ratio was normalized to baseline (T0) to compare any fold change variation.

- **Cell Viability & Cytotoxicity**

Cell viability and cytotoxicity were measured using the MTS-based CellTiter 96® assay (Promega, Madison, WI, US) and the TOX7 kit (Merck), respectively, following the manufacturer's instructions.
Collected absorbances at 450 and 490 nm, respectively, were normalized to baseline (T0) to compare any variation in terms of fold change.

Clinical study

Primary PCI and Medical Therapy

All patients received IV aspirin (250 mg) and heparin (70 U/kg) before PPCI. A P2Y12 inhibitor (prasugrel or ticagrelor) was mandatory, with long-term aspirin maintenance. Additional medications—including GP IIb/IIIa inhibitors, ACE inhibitors or ARBs, β-blockers, statins, and mineralocorticoid receptor antagonists—were prescribed per guideline recommendations.
Infarct-related artery flow was assessed using TIMI criteria pre- and post-revascularization by two independent, blinded cardiologists (G.M. and G.C.).

CMR Acquisition and Analysis

CMR was performed on a 1.5-T Magnetom Avanto scanner (Siemens, Erlangen, Germany) using a standardized post-infarction protocol. This included:

- Cine SSFP sequences (long axis TR/TE 3.5/1.2 ms; short axis 6.0/1.0 ms; slice thickness 6 mm)
- T2-weighted triple inversion-recovery imaging (TR 2×RR, TE 61 ms, TI 160 ms)
- First-pass perfusion and late gadolinium enhancement (LGE) imaging 8–15 minutes after 0.2 mmol/kg Gadobutrol (Gadovist®) injection

Images were analyzed using CVI software (Circle CVI v5.13.7). CMVO was defined as persistent hypoenhancement within infarcted areas. (11) Two experienced readers, blinded to clinical data, independently reviewed all CMR images (F.T. and M.M.).

Echocardiography

TTE was performed using GE Vivid E9/E95 platforms with M5S transducers. LV volumes were calculated by the Simpson biplane method. LVR was defined as an increase in LV end-diastolic volume index (LVEDVI) >20% at 6-month follow-up. (12) Additional assessments included wall motion score index, mitral inflow velocities, deceleration time, and mitral regurgitation grading.

Follow-Up

All patients underwent 6-month TTE follow-up. LVR and MACEs were assessed. For patients with multiple events, only the first was included in endpoint analysis. Follow-up for patients treated elsewhere was completed by phone interviews with patients, physicians, or family members.

Supplementary Results

Preclinical modeling

IgG quantification after isolation

Total IgGs were quantified after isolation from the sera of seropositive STEMI patients and seronegative controls. Table S1 reports the IgG concentration for each sample before its normalization to the final concentration of 3,5 mg/ml, used after 1:20 dilution in disease modeling experiments.

Table S1: IgG concentration after isolation

Subjects	Subject number	Total IgGs (mg/ml)
STEMI	1	4,12
	2	5,25
	3	7,60
	4	4,99
CONTROL	1	7,89
	2	7,94
	3	8,75
	4	6,29

Cell immunophenotype before treatment

hcMVECs were provided as already characterized by the manufacturer. Their endothelial immunophenotype was progressively confirmed during cell expansion by assessing the expression of four markers commonly expressed by differentiated cells: PECAM, VE-cadherin, AT1R, and ETAR. Cells were all positive for PECAM and VE-cadherin, which delineated the cell contours well. AT1R and ETAR were also expressed in a typical receptor punctiform distribution throughout each cell, without any condensed area of expression.
Expression of these endothelial markers (apart from PECAM (CD31)) can be appreciated in the p8 cells used as modeling controls (no treatment, T0) (Figure 1A for VE-cadherin, Figure 4A for AT1R, and Figure 5A for ETAR in the main manuscript).

Cellular alterations observed during treatment

The quantitative observations regarding the cellular alterations are summarized in Table S2. Signs of morphometric changes, endothelial cell activation, mitochondrial dysfunction, and variations in cell survival were analyzed and quantified at 24 and 48 hours. For each quantitative variable, data are expressed as a fold change to T0 (pre-treatment).

Table S2: Quantitative observations regarding cellular alterations

IN VITRO CARDIAC MICROVASCULAR MODELING	CELLULAR PATTERN	CELL TREATMENTS	24h (fold change to T0)	48h (fold change to T0)
MORPHOMETRIC ALTERATIONS	Cell roundness	STEMI IgGs	0,51 ± 0,02 [0,49 – 0,53]	0,48 ±0,02 [0,46 – 0,50]
		STEMI IgGs+Is	0,46 ± 0,01 [0,46 – 0,47]	0,45 ± 0,01 [0,44 – 0,46]
		CTRL IgGs	0,48 ± 0,01 [0,48 - 0,49]	0,47 ± 0,01 [0,46 – 0,48]
		CTRL IgGs+Is	0,45 ± 0,01 [0,44 – 0,45]	0,47 ±0,03 [0,44 – 0,50]
	Cell aspect ratio	STEMI IgGs	1,04 ± 0,04 [0,99 – 1,08]	1,13 ± 0,06 [1,06 – 1,19]
		STEMI IgGs+Is	1,17 ± 0,02 [1,16 – 1,19]	1,24 ± 0,06 [1,18 – 1,30]
		CTRL IgGs	1,10 ± 0,01 [1,09 – 1,11]	1,16 ± 0,08 [1,12 – 1,20]
		CTRL IgGs+Is	1,27 ± 0,03 [1,24 – 1,30]	1,21 ± 0,1 [1,12 – 1,31]
	Nuclear circularity	STEMI IgGs	0,99 ± 0,01 [0,99 – 1,00]	1,01 ± 0,01 [1,01 – 1,02]
		STEMI IgGs+Is	0,98 ± 0,01 [0,98 – 0,99]	1,01 ± 0,01 [1,01 -1,02]
		CTRL IgGs	0,98 ± 0,01 [0,97 – 0,99]	1,00 ± 0,01 [1,00 – 1,01]
		CTRL IgGs+Is	0,98 ± 0,01 [0,98 – 0,99]	1,01 ± 0,01 [1,01 -1,02]
	Nuclear aspect ratio	STEMI IgGs	1,01 ± 0,01 [1,00 – 1,02]	0,98 ± 0,02 [0,97 -1,00]
		STEMI IgGs+Is	0,98 ±0,01 [0,98 – 0,99]	0,97 ± 0,01 [0,96 – 0,97]
		CTRL IgGs	1,00 ± 0,01 [0,99 -1,00]	0,98 ± 0,01 [0,98 – 0,99]
		CTRL IgGs+Is	1,01 ± 0,01 [0,99 – 1,02]	0,96 ± 0,01 [0,96 – 0,97]
ENDOTHELIAL CELL ACTIVATION	VCAM positivity	STEMI IgGs	3,54 ± 0,45 [3,01 - 3,99]	2,87 ± 0,23 [2,64 – 3,10]
		STEMI IgGs+Is	3,33 ± 0,68 [2,85 - 3,81]	2,56 ± 0,22 [2,33 – 2,78]
		CTRL IgGs	0,92 ± 0,38 [0,64 – 1,20]	4,37 ± 0,5 [3,86 – 4,87]
		CTRL IgGs+Is	1,80 ± 0.07 [1,72 - 1,87]	2,3 ± 0,26 [2,04 – 2,56]
MITOCHONDRIAL DYSFUNCTION	MR positivity	STEMI IgGs	4,49 ± 0,40 [4,09 – 5,04]	2,13 ± 0,87 [1,13 - 3,14]
		STEMI IgGs+Is	2,77 ± 1,23 [1,09 – 4,12]	0,9 ± 0,34 [0,44 – 1,33]
		CTRL IgGs	1,11 ± 0,33 [0,79 – 1,51]	1,50 ± 0,64 [0,84 – 2,29]
		CTRL IgGs+Is	1,88 ± 0,90	1,02 ± 0,10

			[0,68 – 2,41]	[0,88 – 1,14]
CELL SURVIVAL VARIATIONS	Cell viability	STEMI IgGs	1,79 ± 0,11<br>[1,65 – 1,93]	1,49 ±0,05<br>[1,43 – 1,55]
		STEMI IgGs+Is	1,78 ± 0,13<br>[1,60 – 1,89]	1,46 ± 0,05<br>[1,40 - 1,53]
		CTRL IgGs	1,72 ± 0,07<br>[1,63 – 1,81]	1,44 ± 0,06<br>[1,38 – 1,52]
		CTRL IgGs+Is	1,51 ± 0,06<br>[1,43 – 1,58]	1,44 ± 0,07<br>[1,35 – 1,53]
	Cytotoxicity	STEMI IgGs	0,94 ± 0,18<br>[0,56 – 1,12]	0,97 ± 0,1<br>[0,82 – 1,09]
		STEMI IgGs+Is	1,10 ± 0,08<br>[0,99 – 1,16]	0,5 ± 0,09<br>[0,42 – 0,62]
		CTRL IgGs	0,72 ± 0,16<br>[0,52 – 0,87]	0,77 ± 0,10<br>[0,69 – 0,93]
		CTRL IgGs+Is	0,94 ± 0,03<br>[0,90 – 0,98	0,21 ± 0,15<br>[0,11 – 0,45]

STEMI IgGs: double seropositive immunoglobulins from STEMI patients; CTRL IgGs: double negative immunoglobulins from healthy blood donors; Is: pharmacological receptor blockers; MR: MitosoxRed.

Clinical Study

Demographic and clinical features according to autoantibody serostatus are summarized in Table S3. In patients with STEMI, antibody seropositivity — particularly double seropositivity for AT1R-AAs and ETAR-AAs — was independently associated with an increased risk of adverse myocardial and clinical outcomes. In the studied cohort of 80 patients, as reported in Table S3, the CMVO, LVR, and MACE increased stepwise across antibody burden: CMVO occurred in 22.9% of seronegative, 40.0% of single-positive, and 80.0% of double-positive patients ($p<0.001$), LVR was observed in 11.4%, 32.0%, and 70.0% respectively ($p<0.001$) and MACE occurred in 8.6%, 20.0%, and 55.0% respectively ($p=0.001$).

Importantly, in multivariable logistic regression models adjusting for key clinical variables (age, sex, comorbidities, infarct location), double antibody seropositivity remained a significant independent predictor of all three outcomes. For CMVO, double positivity was associated with an odds ratio (OR) of 10.64 (95% CI: 2.17–18.17, $p=0.004$), for LVR, OR = 8.71 (95% CI: 1.84–16.33, $p=0.007$) and for MACE, OR = 7.66 (95% CI: 1.55–12.84, $p=0.012$)

Single seropositivity, while associated with intermediate event rates in descriptive analysis, did not reach statistical significance in adjusted models for any outcome.

These findings suggest that autoimmune stimulation via GPCR-targeting antibodies may play a pivotal role in the pathophysiology of post-infarction injury. The progressive increase in adverse events across serostatus categories further supports the dose-dependent pathologic relevance of antibody burden and highlights the potential utility of antibody profiling in post-STEMI risk stratification.

Table S3: Demographic and clinical characteristics of patients with autoantibodies double seronegativity, single, or double seropositivity

Characteristic	All Patients (n=80)	Double seronegative (n=35)	Single seropositivity (n=25)	Double seropositivity (n=20)	p
Age, years	59 (54–69)	58 (52–68)	60 (55–70)	61 (56–71)	0.420
Male sex, n (%)	68 (85.0)	29 (82.9)	22 (88.0)	17 (85.0)	0.850
Smoking, n (%)	33 (41.3)	15 (42.9)	10 (40.0)	8 (40.0)	0.980
Diabetes, n (%)	11 (13.8)	4 (11.4)	3 (12.0)	4 (20.0)	0.590
Hypertension, n (%)	36 (45.0)	14 (40.0)	10 (40.0)	12 (60.0)	0.310
Dyslipidemia, n (%)	25 (31.3)	10 (28.6)	8 (32.0)	7 (35.0)	0.840
Anterior MI, n (%)	48 (60.0)	21 (60.0)	15 (60.0)	12 (60.0)	1.000
Pain-to-balloon time, min	190 (120–390)	200 (130–400)	180 (120–370)	175 (110–350)	0.510
Post-PCI TIMI 3 flow, n (%)	70 (87.5)	32 (91.4)	22 (88.0)	16 (80.0)	0.460
LVEDVI, mL/m²	56 (48–65)	55 (47–64)	57 (50–66)	58 (52–68)	0.430
LVEF, %	50 (43–56)	51 (45–57)	49 (42–55)	47 (41–53)	0.380
WMSI	1.60 (1.35–1.90)	1.55 (1.30–1.85)	1.65 (1.40–1.95)	1.75 (1.50–2.00)	0.310
History of CAD, n (%)	6 (7.5)	3 (8.6)	2 (8.0)	1 (5.0)	0.900
Troponin at admission, ng/L	3500 (280–11000)	3200 (250–10000)	3600 (300–11500)	4000 (350–12000)	0.440
Peak troponin, ng/L	77000 (43000–190000)	68000 (41000–170000)	79000 (46000–200000)	85000 (50000–220000)	0.390
**CMVO, n (%)**	34 (42.5)	8 (22.9)	10 (40.0)	16 (80.0)	**<0.001**
**LVR, n (%)**	26 (32.5)	4 (11.4)	8 (32.0)	14 (70.0)	**<0.001**
**MACE, n (%)**	19 (23.8)	3 (8.6)	5 (20.0)	11 (55.0)	**0.001**

CAD, coronary artery disease; CMVO, coronary microvascular obstruction; LVEDVI, left ventricle end-diastolic volume index; LVEF, left ventricle ejection fraction; LVR, left ventricle remodeling; MACE, major adverse cardiovascular events; PCI, percutaneous coronary intervention; WMSI, wall motion score index.

To further quantify the magnitude and biological relevance of the observed differences, effect size estimates for key in vitro and clinical outcomes are reported in **Table S4 and S5.**

Table S4. Effect size estimates for major in vitro endpoints in the cardiac microvascular endothelial cell model.

	Time point	Comparison	Effect size
**Cell morphometry**	Cell roundness (24 h)	STEMI IgGs vs CTRL IgGs	≈ 1.7
	Cell aspect ratio (24 h)	STEMI IgGs vs CTRL IgGs	≈ −1.9
	Nuclear circularity (24 h)	STEMI IgGs vs CTRL IgGs	≈ 0.6
	Nuclear aspect ratio (24 h)	STEMI IgGs vs CTRL IgGs	≈ 0.7
**Endothelial activation**	VCAM-1 positivity (24 h)	STEMI IgGs vs CTRL IgGs	≈ 5.6
**Mitochondrial dysfunction**	MitoSOX™ positivity (24 h)	STEMI IgGs vs CTRL IgGs	≈ 8.6
**Cell survival**	Cell viability (48 h)	STEMI IgGs vs CTRL IgGs	≈ 0.9
**Cytotoxicity**	LDH release (48 h)	STEMI IgGs vs STEMI IgGs + inhibitors	≈ 4.8
**Receptor localization**	AT1R / ETAR redistribution	STEMI IgGs vs CTRL IgGs	—

Effect size estimates for the main quantitative in vitro endpoints comparing human cardiac microvascular endothelial cells exposed to IgGs from double-seropositive STEMI patients versus seronegative controls, and to receptor blockade where applicable. Effect sizes are reported as Hedges' g (bias-corrected standardized mean differences) to account for small sample size. Magnitude was interpreted as small (~0.2), moderate (~0.5), or large (≥0.8). Receptor expression and localization were assessed qualitatively and therefore not assigned a standardized effect size.

Table S5: Effect size estimates for clinical outcomes associated with autoantibody serostatus

Outcome	Comparison	Effect size metric	Effect size (95% CI)	Magnitude
**CMVO**	Double-positive vs seronegative	Odds ratio	10.64 (2.17–18.17)	Very large
**LVR**	Double-positive vs seronegative	Odds ratio	8.71 (1.84–16.33)	Very large
**MACE**	Double-positive vs seronegative	Odds ratio	7.66 (1.55–12.84)	Very large

Effect size estimates for major clinical outcomes according to autoantibody serostatus in the STEMI cohort. Effect sizes are reported as odds ratios derived from multivariable logistic regression models, adjusted for relevant clinical covariates, and are presented with 95% confidence intervals. CMVO indicates coronary microvascular obstruction; LVR, left ventricular remodeling; MACE, major adverse cardiovascular events.

To provide full transparency of the multivariable analysis, the detailed results of the stepwise logistic regression model for CMVO, including adjusted odds ratios and 95% confidence intervals for all variables retained in the final model, are reported in Supplementary Table S6.

Table S6. Multivariable logistic regression analysis for predictors of coronary microvascular obstruction (CMVO)

Variable	Adjusted OR	95% CI	p value
Autoantibody positivity (single or double)	3.01	1.21-6.51	**0.011**
Age (per year)	0.97	0.93-1.05	0.621
Male sex	0.85	0.22-2.89	0.785
Anterior myocardial infarction	1.95	1.08-2.01	**0.021**
Ischemic time (per 60 min)	1.02	1.01-1.04	**0.013**
Diabetes mellitus	0.54	0.39-3.54	0.527

Sensitivity analyses			
Infarct size	1.22	1.06-1.78	0.08
Hypertension	0.51	0.44-3.54	0.411
Smoking	0.78	0.69-2.11	0.529
Dyslipidaemia	2.52	0.99-7.11	0.817

The primary model included age, sex, ischemic time, infarct location, diabetes mellitus, and autoantibody positivity. Infarct size was not included in the primary model because it may represent a downstream marker of microvascular injury; it was evaluated in sensitivity analyses together with hypertension, hypercholesterolemia, and smoking. Results are reported as adjusted odds ratios (OR) with 95% confidence intervals (CI)

Supplementary Figures

Supplementary Figure 1S. Morphological effects of patient-derived IgGs on human cardiac microvascular endothelial cells (hcMVECs). Representative phase-contrast images show hcMVECs under different conditions. Untreated cells (A, T0) displayed typical cobblestone morphology. Exposure to IgGs from STEMI patients (B, T24; C, T48) induced size reduction, consistent with cytoarchitecture injury. Receptor antagonism with valsartan and bosentan (D, T24; E, T48) induced elongated morphology. In contrast, IgGs from seronegative controls preserved baseline features (F, T24; G, T48), with further elongation under receptor blockade (H, T24; I, T48). Scale bar = 50 μm.

References

1. Tona F, Civieri G, Vadori M, Masiero G, Iop L, Marra MP, et al. Association of angiotensin II receptor type 1 and endothelin-1 receptor type A agonistic autoantibodies with adverse remodeling and cardiovascular events after acute myocardial infarction. *J Am Heart Assoc*. 2024;13:e032672.
2. Wallukat G, Homuth V, Fischer T, Lindschau K, Horstkamp B, Juepner A, Baur E, Nissen E, Vetter K, Neichel D, Dudenhausen JW, Haller H, Luft FC. Patients with preeclampsia develop agonistic autoantibodies against the angiotensin AT1 receptor. *J Clin Invest.* 1999;103:945-952.
3. Tway TM, Shlykov SJ, Day M-C, Sanborn BM, Gilstrap LC, Xia Y, Kellems RE. Antibodies from preeclamptic patients stimulate increased intracellular Ca2+ mobilization through angiotensin receptor activation. *Circulation* 2004;110:1612-1619.
4. Yang X, Wang F, Chang H, Zhang S, Yang L, Wang X, Cheng X, Zhang M, Ma XL, Liu H. Autoantibody against AT1 receptor from preeclamptic patients induces vasoconstriction through angiotensin receptor activation. *J Hypertens* 2008;26:1629-1635.
5. Ferron L, Capuano V, Ruchon Y, Deroubaix E, Coulombe A, Renaud J-F. Angiotensin II signaling pathways mediate expression of cardiac T-type calcium channels. *Circ. Res.* 2003;93:1241-48. DOI: 10.1161/01.RES.0000106134.69300.B7.
6. Kill A, Tabeling C, Undeutsch R, Kuehl AA, Guenther J, Radic M, O Becker M, Heidecke H, Worm M, Witzenrath M, Burmester G-R, Dragun D, Riemekasten G. Autoantibodies to angiotensin and endothelin receptors in systemic sclerosis induce cellular and systemic events associated with disease pathogenesis. *Arthritis* 2014;16:R29. DOI: 10.1186/ar4457.
7. Li R, Mi X, Yang S, Yang Y, Zhang S, Hui R, Chen Y, Zhang W. Long-term stimulation of angiotensin II induced endothelial senescence and dysfunction. *Experimental Gerontology* 2019;119:212-220. DOI: 10.1016/j.exger.2019.02.012.

8. Schindelin J, Arganda-Carreras I, Frise E, Kaynig V, Longair M, Pietzsch T, et al. Fiji: An open-source platform for biological-image analysis. *Nat Methods*. 2012;9:676–82
9. Ashok P and Alizadeh E. Cell form and function: interpreting and controlling the shape of adherent cells. *Trends in Biotechnology* 2019;37(4):347-57.
10. Selig M, Azizi S, Walz K, et al. Cell morphology as a biological fingerprint of chondrocyte phenotype in control and inflammatory conditions, *Front Immunol* 2023;14:1102912.
11. Demirkiran A, Everaars H, Amier RP, et al. Cardiovascular magnetic resonance techniques for tissue characterization after acute myocardial injury. *Eur Heart J Cardiovasc Imaging* 2019;20:723–734.
12. Bolognese L, Neskovic AN, Parodi G, et al. Left ventricular remodeling after primary coronary angioplasty: patterns of left ventricular dilation and long-term prognostic implications. *Circulation*. 2002 Oct 29;106(18):2351-7. doi: 10.1161/01.cir.0000036014.90197.fa.
