# Supplementary figures and images for: Functional autoantibodies and coronary microvascular obstruction in STEMI: a translational link between immune mechanisms and prognostic outcomes
Source: Front Cardiovasc Med. 2026 Feb 25;13:1739236. doi: 10.3389/fcvm.2026.1739236 (PMC12977973; doi:10.3389/fcvm.2026.1739236)

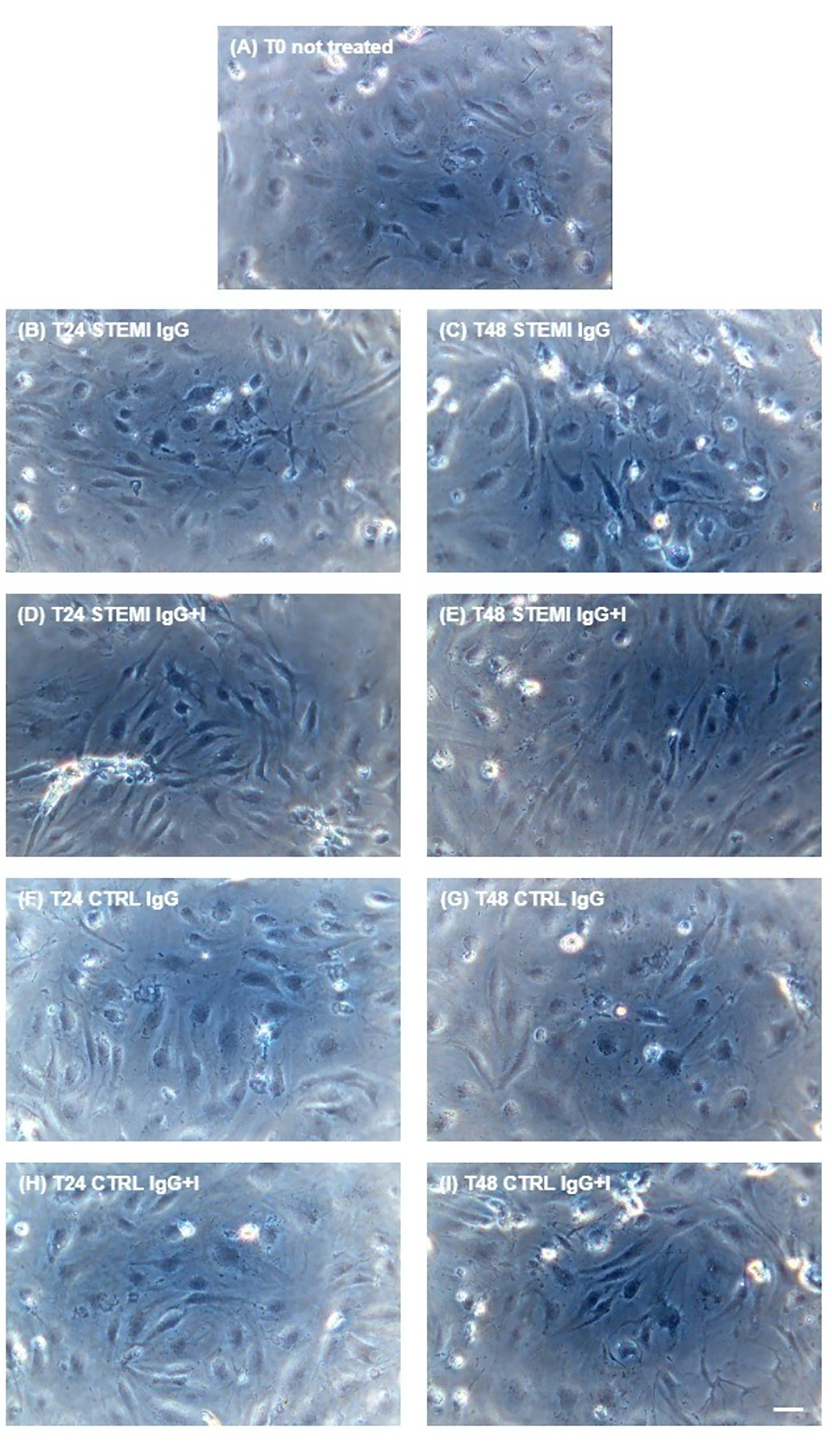

Supplement: Supplementary file 2 [file Image1.tif]
